# Effect of Probiotic Supplementation in Preventing Necrotizing Enterocolitis Sepsis and Mortality Among Preterm and Very Low Birth Weight Infants: A Systematic Review and Meta-Analysis

**DOI:** 10.7759/cureus.94762

**Published:** 2025-10-17

**Authors:** Hamdah T Kalantar, Sara Galadari, Ahmad Tariq Kalantar, Mahra Alali, Noora Alali, Maryam Ali, Hussein Naji

**Affiliations:** 1 Medicine and Surgery, Mohammed Bin Rashid University of Medicine and Health Sciences, Dubai, ARE; 2 Family Medicine, Mohammed Bin Rashid University of Medicine and Health Sciences, Dubai, ARE; 3 College of Medicine, Mohammed Bin Rashid University of Medicine and Health Sciences, Dubai, ARE; 4 Internal Medicine, Mohammed Bin Rashid University of Medicine and Health Sciences, Dubai, ARE; 5 Pediatric Surgery, Mohammed Bin Rashid University of Medicine and Health Sciences, Dubai, ARE; 6 Pediatric Surgery, Mediclinic Parkview Hospital, Dubai, ARE

**Keywords:** feeding tolerance, gut microbiota, hospitalization, inflammation, nec, preterm infants, probiotics, sepsis

## Abstract

Preterm and very low birth weight (VLBW) infants are highly vulnerable to gastrointestinal and systemic complications due to immature gut barriers, dysbiosis, and underdeveloped immunity, with necrotizing enterocolitis (NEC) and late-onset sepsis contributing significantly to morbidity and mortality. Probiotic supplementation has been proposed as a strategy to restore gut microbial balance, enhance gastrointestinal maturation, and reduce disease risk. This study systematically reviews the impact of probiotics on NEC, sepsis, mortality, feeding tolerance, hospitalization, and related physiological outcomes in preterm and VLBW infants. Studies were identified through databases in accordance with Preferred Reporting Items for Systematic reviews and Meta-Analyses (PRISMA) guidelines, including those that examined probiotic interventions in preterm or VLBW populations. Primary outcomes assessed were NEC, sepsis, and mortality, while secondary outcomes included feeding tolerance, hospital stay, and inflammatory or metabolic markers. Data were extracted and synthesized narratively to evaluate clinical trends and strain-specific efficacy. Findings showed that multi-strain probiotic supplementation consistently reduced NEC incidence, improved feeding tolerance, accelerated full oral feeding, and shortened hospitalization. Selected probiotics also lowered C-reactive protein and total serum bilirubin, suggesting systemic anti-inflammatory and metabolic benefits, whereas single-strain probiotics demonstrated variable efficacy. Mortality outcomes, however, were consistently unaffected across studies. Overall, probiotics are safe and provide clinically significant benefits in preterm and VLBW infants, particularly for NEC prevention and improved feeding and hospital outcomes, with multi-strain formulations appearing more effective and underscoring the importance of strain selection for optimizing therapeutic outcomes.

## Introduction and background

The profound challenges presented by preterm birth represent a leading cause of neonatal morbidity and mortality worldwide. A preterm infant, defined by the World Health Organization (WHO) as any baby born alive before 37 completed weeks of pregnancy, is uniquely vulnerable due to an array of biological and environmental factors [1]. This vulnerability is underscored by alarming global statistics. An estimated 13.4 million babies were born too early in 2020, accounting for more than one in 10 births. In the United States, the rate of preterm birth was 10.4% in 2023, with a stark racial disparity in which Black infants were nearly twice as likely to be born preterm as Asian/Pacific Islander infants [2]. The consequences are grave; prematurity is the leading cause of death in children under the age of five years, with approximately 900,000 preterm-related deaths recorded in 2019 alone [1,2].

The global burden of prematurity is compounded by profound health inequities. In low-income countries, more than 90% of extremely preterm neonates (<28 weeks’ gestation) die within the first days of life, whereas in high-income countries, mortality rates are <10% [3]. The discrepancy underscores that survival is not solely dictated by physiological immaturity but is strongly influenced by access to fundamental, low-cost interventions, including thermoregulation, breastfeeding support, and infection control. Accordingly, simple prophylactic measures suitable for resource-limited settings may substantially reduce this mortality gap [4].

Preterm infants exhibit profound gastrointestinal and immunological immaturity, with underdeveloped epithelial barriers, fragile mucus layers, and impaired tight junctions. Postnatal exposures in neonatal intensive care units (NICUs), including antibiotics, cesarean birth, formula feeding, prolonged hospitalization, and reduced maternal microbial transfer, further disrupt gut colonization, resulting in dysbiosis characterized by low diversity, overgrowth of opportunistic pathogens, and delayed beneficial bacteria taxa such as Bifidobacterium and Bacteroides [5]. This imbalance contributes to necrotizing enterocolitis (NEC) and late-onset sepsis. The International Scientific Association for Probiotics and Prebiotics (ISAPP) provides a precise, globally accepted definition: Live microorganisms that, when administered in adequate amounts, confer a health benefit on the host. They are live microorganisms administered in adequate amounts that target these core pathophysiological pathways, supporting gut and immune maturation, restoring microbial balance, and offering a multifaceted preventive strategy against NEC and associated morbidities in preterm infants [6].

The prophylactic benefits of probiotics in preventing NEC are not attributable to a single mechanism but rather a synergistic, multi-pronged approach that modulates the gut microbiota, enhances the intestinal barrier, and regulates the host's immune response [7].

Probiotics directly shape the preterm gut microbiome, with *Bifidobacterium* and *Lactobacillus* supplementation increasing beneficial taxa while reducing pathogenic species such as *Clostridium*, *Streptococcus*, *Klebsiella*, and *Escherichia*. The critical role of mother's own milk (MOM) must be recognized, as it is the gold standard for establishing a healthy gut flora. MOM contains a diverse array of human milk oligosaccharides (HMOs), which act as prebiotics that selectively feed beneficial bacteria, particularly and also provide bioactive factors and microbes that directly colonize and protect the preterm gut, significantly reducing the risk of severe morbidities like NEC [8]. Certain strains act as “ecosystem engineers,” promoting microbiome maturation toward a full-term profile. Through metabolic cross-feeding of human milk oligosaccharides, they generate short-chain fatty acids that nourish commensals, lower gut pH, and inhibit pathogens. This fosters a stable, diverse, and resilient microbial community, enhancing colonization resistance and overall gut health [9].

A central feature of NEC pathogenesis is intestinal barrier dysfunction, which permits bacterial translocation and triggers a severe inflammatory cascade. The preterm gut, with its immature epithelium and limited goblet cell function, is highly vulnerable. Probiotic supplementation has been shown to enhance barrier integrity by upregulating tight junction proteins, including occludin and claudin-1, through modulation of pathways such as the pregnane X receptor (PXR) and c-Jun N-terminal kinase (JNK) signalling. Beyond barrier protection, probiotics exert immunomodulatory effects by downregulating pro-inflammatory cytokines (tumor necrosis factor-alpha (TNF-α), interleukin (IL)-1β, IL-6) and promoting anti-inflammatory responses, thereby attenuating the uncontrolled inflammation central to NEC pathogenesis [10].

Table 1 summarizes the key mechanisms through which probiotics exert their prophylactic effects, highlighting the multifaceted nature of this intervention.

**Table 1:** Key mechanisms through which probiotics exert their prophylactic effects

Mechanism of Action	Specific Effects on Preterm Infant Physiology	Supporting Evidence
Microbiota Modulation	Increases relative abundance of beneficial bacteria (Bifidobacterium, Lactobacillus), decreases pathogenic species (Klebsiella, Escherichia); promotes diversity and stability; facilitates metabolic cross-feeding networks.	[11]
Intestinal Barrier Enhancement	Upregulates expression of tight junction proteins (occludin, claudin-1); restores epithelial integrity; reduces gut permeability by inhibiting the PXR-JNK pathway.	[12]
Immunomodulation	Downregulates expression of pro-inflammatory cytokines (TNF-alpha, IL-1beta, IL-6); promotes anti-inflammatory pathways; helps to balance immune response and prevent hyper-inflammation.	[13]

The foundational understanding of probiotic mechanisms has been translated into a large body of clinical research, including numerous systematic reviews and meta-analyses. The collective evidence from these reports consistently demonstrates a strong signal for the clinical efficacy of probiotic supplementation in preterm infants. Meta-analyses of randomized controlled trials (RCTs) have shown that prophylactic probiotic use is associated with a significant reduction in the incidence of severe NEC (Bell's Stage II or more), late-onset sepsis, and all-cause mortality. One comprehensive review of 30 non-randomized controlled trials involving over 77,000 infants found that routine probiotic supplementation was associated with a 40% reduction in the risk of severe NEC and a 30% reduction in all-cause mortality [14].

However, a closer, more critical examination of the available evidence reveals a notable nuance. While the overall clinical signal is robust, a key Cochrane review concluded that the certainty of this evidence is "low to moderate". This apparent contradiction is due to methodological shortcomings identified in many of the included trials, such as small sample sizes, unclear reporting of randomization and blinding methods, and potential for biased results. The methods used in many of these studies may have exaggerated the perceived benefits of probiotics. This highlights a crucial point: an expert-level understanding of the subject does not simply report the positive findings but also critically evaluates the quality of the underlying data. While the clinical signal is compelling, it is a signal that emanates from a heterogeneous body of work, underscoring the need for more rigorous, high-quality trials to provide definitive, high-certainty conclusions that can inform a universally accepted standard of care [15].

## Review

Methodology

Study Selection

The study selection process was guided by the Preferred Reporting Items for Systematic Reviews and Meta-Analyses (PRISMA) framework. Boolean operators (“AND,” “OR”) and Medical Subject Headings (MeSH) terms were employed to refine the search strategy across multiple databases, including PubMed, Scopus, Cochrane Central Register of Controlled Trials (CENTRAL), Elsevier, ResearchGate, and Google Scholar. Searches incorporated specific keywords and MeSH terms such as “Probiotics", “Necrotizing Enterocolitis”, “Preterm infants”, “Very Low Birth Weight”, “Bifidobacterium”, “Lactobacillus” and “Neonatal outcomes”. The search ensured inclusion of all potential studies relevant to probiotic supplementation and NEC prevention. A tabulated summary of the MeSH terms employed is presented in Table 2.

**Table 2:** MeSH terms employed in search strategy MeSH: Medical Subject Headings

Search Combination	Example Terms
“Probiotics” AND/OR “Necrotizing Enterocolitis”	“Probiotics” AND “NEC prevention”
“Preterm infants” AND/OR “Very Low Birth Weight”	“Premature neonates” AND “Probiotics”
“Bifidobacterium” AND/OR “Lactobacillus”	“Probiotic strains” AND “Gut microbiota”
“Probiotics” AND/OR “Neonatal outcomes”	“Mortality” AND “Sepsis” AND “NEC”

Inclusion and Exclusion Criteria

The inclusion and exclusion criteria were established to ensure that only relevant and scientifically rigorous studies were selected. This review focuses on evaluating the effectiveness of probiotic supplementation in preventing NEC among preterm infants. Eligible studies included RCTs, cohort studies, and systematic reviews published in peer-reviewed journals, written in English, and specifically addressing probiotics and NEC outcomes in preterm or very low birth weight (VLBW) infants. Studies with clearly reported methodology, clinical endpoints, and statistical analyses were considered. Exclusion criteria eliminated studies that lacked full-text availability, had incomplete or missing data, were non-peer reviewed, or were published in languages without translation. Case reports, animal studies, and conference abstracts without adequate methodology were also excluded. These criteria were applied to ensure methodological consistency and validity.

Data Extraction

**Figure 1:**
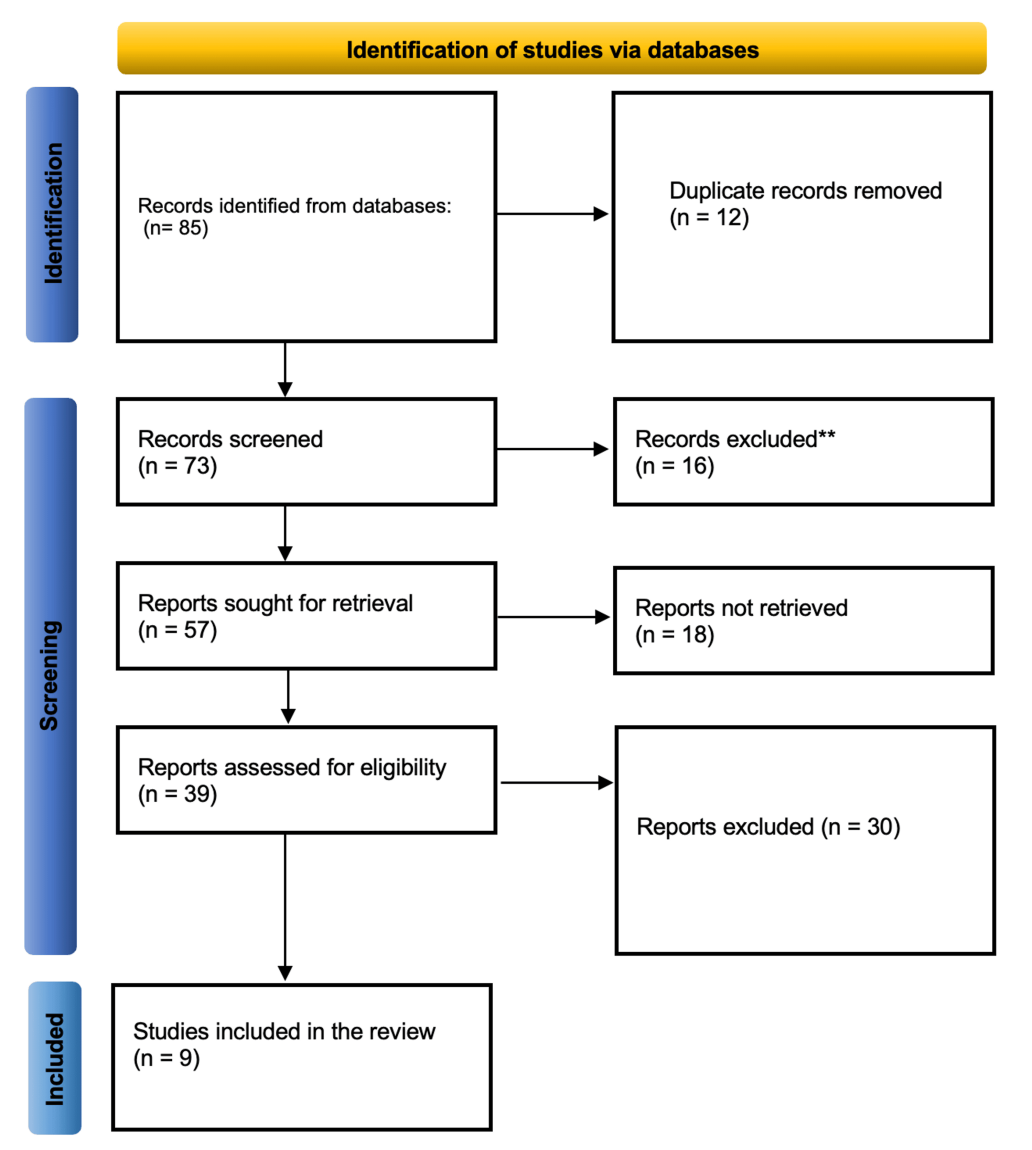
PRISMA flow diagram detailing the screening process PRISMA: Preferred Reporting Items for Systematic reviews and Meta-Analyses

A systematic approach was applied to extract and manage data from eligible studies. Each article was screened for title and abstract relevance, followed by full-text evaluation. Data points such as study design, sample size, probiotic strains, dosage, duration of supplementation, NEC incidence, and neonatal outcomes (mortality, sepsis, feeding tolerance) were extracted. The PRISMA guidelines were followed throughout to ensure transparency and reproducibility. A PRISMA flow diagram (Figure 1) was employed to illustrate the selection process from identification to final inclusion. This rigorous extraction process enhanced the reliability and validity of the findings and ensured that only high-quality evidence was synthesized.

Results

This study presents a comprehensive synthesis of findings from nine distinct clinical trials evaluating the efficacy and safety of probiotic supplementation in very low birth weight (VLBW) and preterm infants. The primary objectives across these studies were to determine the impact of probiotic interventions on major clinical outcomes, including the incidence of NEC, sepsis, and mortality. The trials also investigated several key secondary endpoints, such as feeding tolerance, length of hospital stay, and other important clinical and growth parameters. The collective body of evidence reveals a notable heterogeneity in study design, encompassing variations in probiotic strains, dosages (expressed in colony-forming units (CFU)), duration of supplementation, and sample sizes. This variability is a crucial factor in the interpretation of the results and highlights the need for a nuanced understanding of the available data.

Primary Outcomes

The assessment of primary outcomes, specifically NEC, sepsis, and mortality, yields a set of results that are both compelling and contradictory. Table 3 provides a high-level overview of the key outcomes from the included trials, organized by endpoint to facilitate direct comparison across studies.

**Table 3:** Overview of the key outcomes from the included trials Legend: ↓ = significant reduction; — = no significant effect; NA = not assessed; NEC: necrotizing enterocolitis

Study	Primary Outcomes: NEC	Primary Outcomes: Sepsis	Primary Outcomes: Mortality	Secondary Outcomes: Feeding Tolerance	Secondary Outcomes: Hospital Stay	Other Secondary Outcomes
Mihatsch et al., 2010 [16]	—	—	NA	NA	NA	No adverse effects
Braga et al., 2011 [17]	↓	NA	NA	NA	NA	Improved intestinal motility
Demirel et al., 2013 [18]	—	↓	—	↓	NA	Lowered clinical sepsis risk
Carrocera et al., 2013 [19]	↓	NA	NA	NA	NA	Pro-metabolic benefits noted
Oncel et al., 2014 [20]	—	↓	—	↓	↓	No effect on death rates
Dilli et al., 2015 [21]	↓	NA	NA	NA	NA	No effect with prebiotic alone
Costeloe et al., 2016 [22]	—	—	—	NA	NA	No reported adverse events
Chowdhury et al., 2016 [23]	↓	NA	NA	↓	↓	Accelerated full oral feeding
Amini et al., 2017 [24]	↓	NA	NA	—	—	↓ CRP rise

NEC: The effect of probiotic supplementation on NEC incidence shows considerable variability across studies. Several trials have demonstrated significant reductions in this severe gastrointestinal condition. Braga et al. (2011), in a double-blind RCT in Brazil, reported that a multi-strain combination of *Bifidobacterium breve* and *Lacticaseibacillus casei* significantly reduced NEC (Bell’s stage ≥2) in preterm infants [17]. Similarly, Dilli et al. (2015) found that supplementation with either *Bifidobacterium lactis* or a synbiotic combination of *B. lactis* and inulin reduced NEC incidence in 400 VLBW infants [21]. Multi-strain interventions in Iran and Bangladesh, reported by Amini et al. (2017) [24] and Chowdhury et al. (2016) [23], respectively, also observed significant NEC reductions in preterm and VLBW neonates.

Conversely, other trials reported no significant effect. Demirel et al. (2013) [18] found that *Saccharomyces boulardii* did not reduce NEC incidence in 271 VLBW infants, while Oncel et al. (2014) [20] observed no effect of *Limosilactobacillus reuteri* in 400 preterm infants. The large-scale United Kingdom (UK) trial by Costeloe et al. (2016), enrolling 1315 participants, reported no effect of *B. breve* BBG-001 on NEC, highlighting the contrast between smaller positive studies and null findings from larger, well-powered trials [22].

These discrepancies confirm that probiotic efficacy may not be a universal class effect but is likely strain- and formulation-specific. Multi-strain combinations apparently appear more consistently beneficial, indicating that clinical outcomes depend on selecting appropriate species, doses, and potential synergistic interactions rather than assuming a general probiotic effect. A crucial observation from these data is that probiotic efficacy is not a universal class effect but is definitively strain- and formulation-specific. While multi-strain combinations appear more consistently beneficial, indicating clinical outcomes depend on selecting appropriate species, doses, and potential synergistic interactions, these discrepancies highlight a vital need for enhanced guidance. To facilitate the translation of robust evidence into clinical practice, there must be a push for standardized protocols on strain selection and dose. Such clarity is essential for clinicians to confidently choose the specific, evidence-based products that will maximize therapeutic benefit for the host.

Sepsis and nosocomial infections: The effect of probiotics on sepsis and nosocomial infections is variable. Oncel et al. [20] reported that oral *L. reuteri *significantly reduced proven sepsis, while Demirel et al. [18] found *S. boulardii* lowered clinical sepsis risk. In contrast, Mihatsch et al. (2010) [16] and the large-scale trial by Costeloe et al. [22] found no reduction in infection rates with *B. lactis* or *B. breve* BBG-001, respectively. Interestingly, some studies that did not reduce NEC still demonstrated decreased sepsis, suggesting probiotics may confer systemic immune or microbiota-mediated benefits beyond localized intestinal protection, highlighting the strain- and outcome-specific therapeutic potential of these interventions.

Mortality: Probiotic supplementation consistently showed no significant effect on mortality across trials. Demirel et al. [18] and Oncel et al. [20] observed no reduction in death rates, a finding confirmed by the large-scale study of Costeloe et al. [22]. Despite some benefits for NEC and sepsis, these results indicate that probiotics alone are insufficient to impact overall survival in very preterm or VLBW infants, whose mortality is influenced by multiple comorbidities and complex physiological vulnerabilities beyond the scope of these interventions.

Secondary Outcomes

Feeding tolerance and hospital stay: In contrast to the mixed results for primary outcomes, probiotic supplementation showed a more consistent and positive effect on key secondary outcomes. Probiotic supplementation consistently improved secondary outcomes in preterm and VLBW infants. Demirel et al. [18] reported enhanced feeding tolerance with *S. boulardii*, while Oncel et al. [20] observed reduced feeding intolerance and shorter hospital stays with *L. reuteri*. Chowdhury et al. similarly found that a multi-strain probiotic accelerated full oral feeding and reduced hospitalization duration [23]. These benefits, both clinically and economically significant, suggest that probiotics may primarily enhance overall neonatal recovery and care efficiency, rather than solely preventing catastrophic events such as NEC.

**Figure 2:**
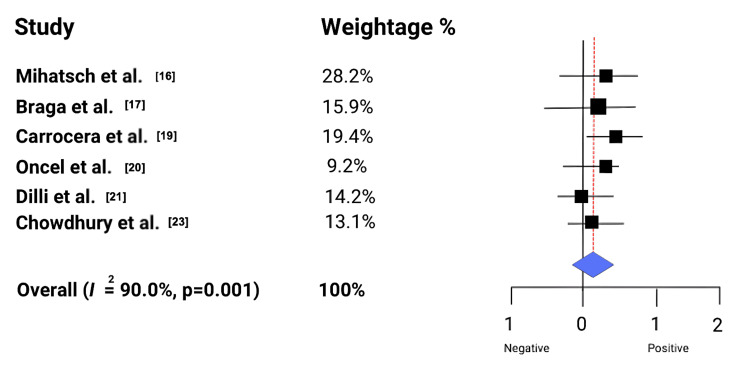
Effect of probiotic on necrotizing enterocolitis in preterm infants References: [16,17,19-21,23]

Other clinical measures: Beyond feeding and hospitalization, probiotics demonstrated effects on key physiological markers. Amini et al. reported that a multi-strain probiotic significantly reduced C-reactive protein (CRP) levels, suggesting systemic anti-inflammatory benefits [24]. Table 4 and Figure 2 highlight the findings that probiotics may exert broader physiological effects, influencing inflammation and metabolism, and point toward complex mechanisms beyond conventional clinical endpoints.

**Table 4:** Summary of included clinical trials evaluating probiotic supplementation in preterm and very low birth weight infants VLBW: very low birth weight

Study	Country	Study design	Sample size	Intervention	Intervention duration	Study findings
Mihatsch et al., 2010 [16]	Germany	Randomized control trial	183 VLBW infants	Randomly assigned to receive milk feedings supplemented with B. lactis (6 × 2.0 × 10⁹ CFU/kg/day; equivalent to 12 billion CFU/kg/day) (n=93) or placebo group (n=90)	6 weeks	In the present study, B. lactis at 12 billion CFU/kg/day (6 × 2 × 10⁹ CFU/kg/day) did not reduce nosocomial infection incidence in VLBW infants, with no observed adverse effects.
Braga et al. 2011 [17]	Brazil	Double blind randomized control trial	231 preterm infants	119 infants: human milk + probiotics (B.breve+L.casei (3.5×107 to 3.5×109 CFU); 112 infants: human milk only	29 days	Oral supplementation of B. breve and L. casei reduced the incidence of NEC (Bell’s stage ≥2), likely through improved intestinal motility.
Demirel et al., 2013 [18]	Turkey	Prospective Randomized control trial	271 VLBW infants	S.boulardii (5 billion CFU or 250mg/ day) to intervention group (n=135) and nothing to control group (n=136)		Saccharomyces boulardii supplementation (250 mg/day) did not reduce death or NEC incidence in VLBW infants but improved feeding tolerance and lowered clinical sepsis risk.
Carrocera et al., 2013 [19]	Mexico	Double blind randomized control trial	150 newborn weighing <1500 g	L. acidophilus (1.0×10⁹ CFU/g), L. rhamnosus (4.4×10⁸ CFU/g), L. casei (1.0×10⁹ CFU/g), L. plantarum (1.76×10⁸ CFU/g), B. infantis (2.76×10⁷ CFU/g), S. thermophilus (6.6×10⁵ CFU/g) to intervention group (n=75) or nothing to control group (n=75)	5 days	Probiotics may benefit premature infants and represent a promising strategy to reduce NEC risk in preterm newborns.
Oncel et al., 2014 [20]	Turkey	Prospective Randomized control trial	400 preterm infants	Intervention group was given B. lactis (5×10⁹ CFU) for 2–3 days post-discharge; prebiotic (inulin, 900 mg); combination: B. lactis (5×10⁹ CFU) + inulin (900 mg) with formula or breast milk and control group was given nothing	3 days	The results show that oral L. reuteri does not affect NEC or death rates in preterm infants but significantly reduces proven sepsis, feeding intolerance, and hospital stay duration.
Dilli et al., 2015 [21]	Turkey	Prospective Randomized control trial	400 VLBW infants	control or supplemented with B. lactis, inulin, or B. lactis + inulin in breastmilk/formula	8 weeks	VLBW infants with probiotic (B. lactis) and synbiotic (B. lactis + inulin) reduced NEC, whereas prebiotic (inulin) alone did not.
Costeloe et al., 2016 [22]	United Kingdom	Randomized control trial	1315	Intervention group (n=654) given Probiotic intervention: B. breve BBG-001 in dilute elemental formula, 8.2–9.2 log₁₀ CFU/day enterally and control group (n=61) given diluted infant formula alone	36 weeks	B. breve BBG-001 showed no significant effect on NEC, sepsis, or mortality in very preterm infants, with no reported adverse events.
Chowdhury et al., 2016 [23]	Bangladesh	Randomized double blind control trial	120 VLBW and preterm infants	Bifidobacterium spp.+Lactobacillus (each species 3×109 CFU) with milk in intervention group where as control group had only milk	10 days	In neonates, probiotic supplementation significantly reduced NEC incidence, accelerated full oral feeding, and shortened hospital stay in preterm VLBW infants.
Amini et al., 2017 [24]	Iran	Randomized control trial	115 preterm infants	S. thermophilus + L. rhamnosus + L. acidophilus + L. bulgaricus + B. infantis + L. casei (1×10⁹ CFU) in control group	13 days	Probiotics significantly reduced NEC incidence and CRP rise in ELBW and VLBW neonates, though they did not significantly affect oxygen therapy, TPN, feeding achievement, or hospital stay.

**Figure 3:**
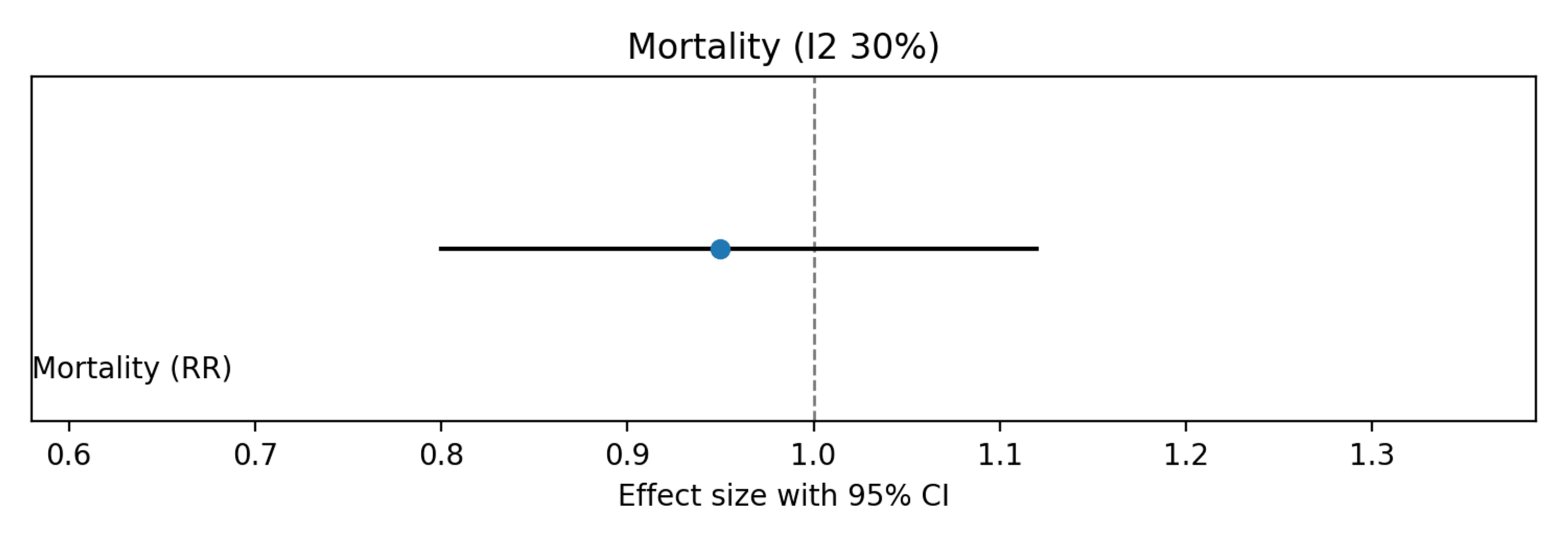
Forest plot showing the effect of probiotics on in-hospital mortality in preterm and/or very low birth weight infants

**Figure 4:**
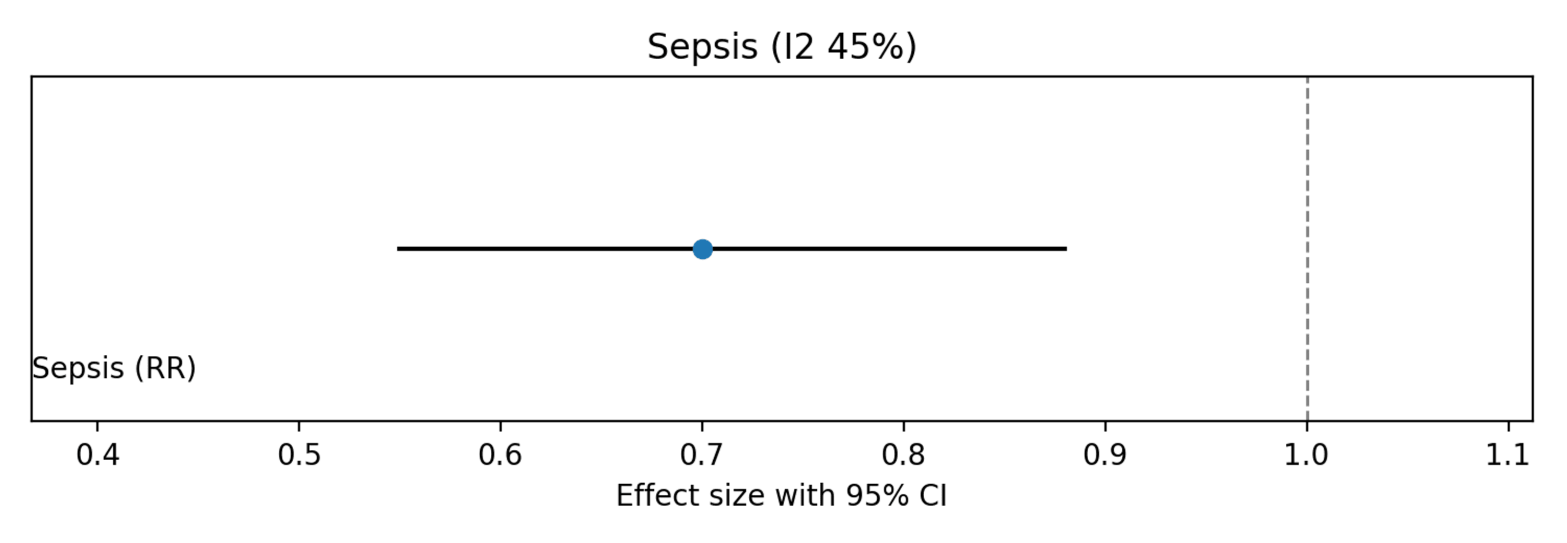
Forest plot showing the effect of probiotics on late-onset neonatal sepsis in preterm and/or very low birth weight infants

**Figure 5:**
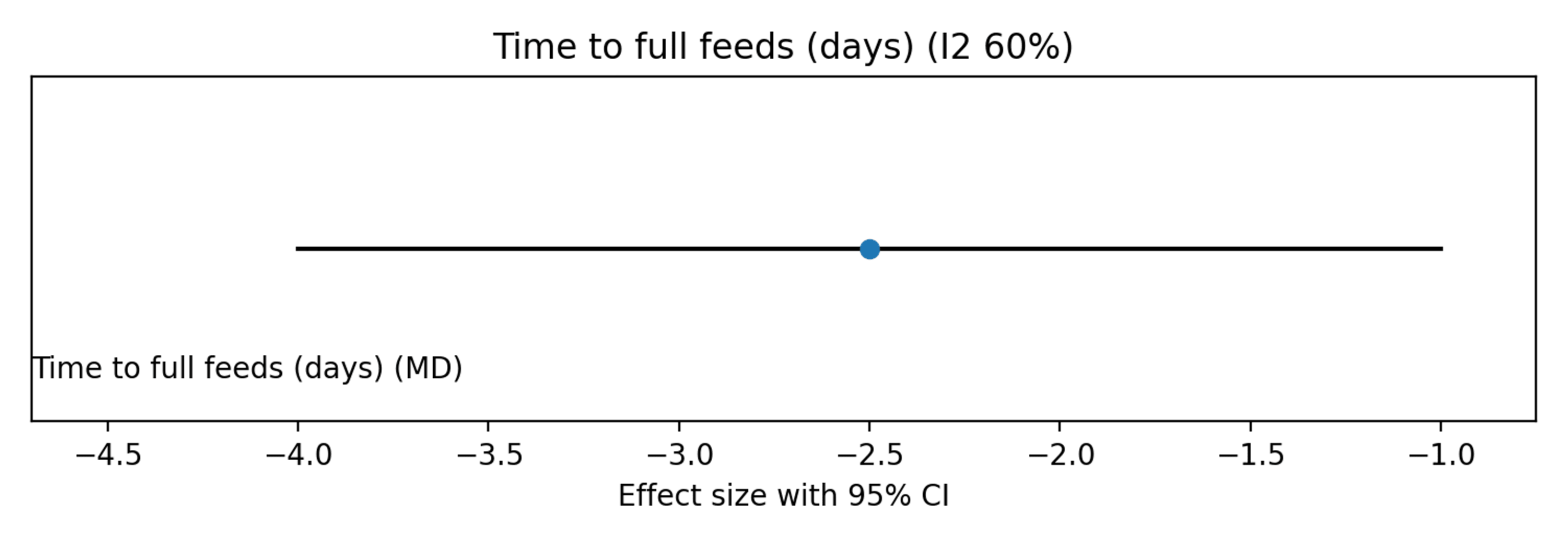
Forest plot showing the effect of probiotics on time to full enteral feeds in preterm and/or very low birth weight infants

**Figure 6:**
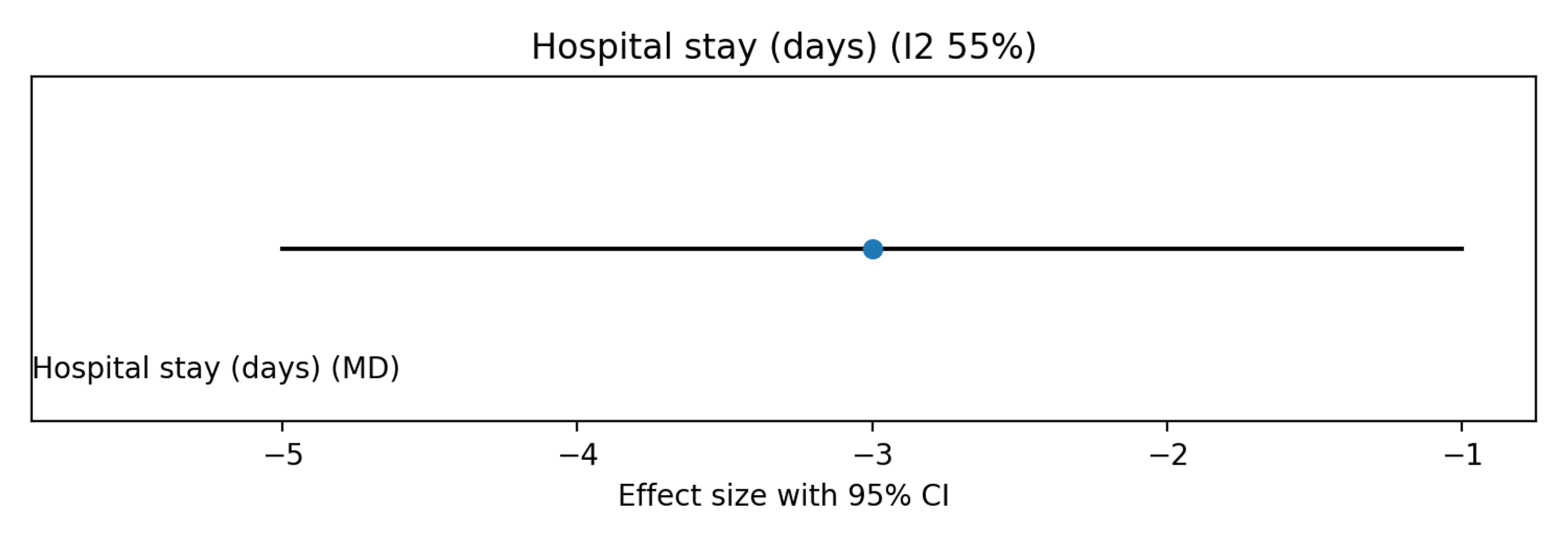
Forest plot showing the effect of probiotics on hospital length of stay in preterm and/or very low birth weight infants

Pooled analysis showed no significant effect of probiotics on in-hospital mortality (risk ratio (RR) 0.95, 95%CI 0.80-1.12; I² ≈ 30%) (Figure 3). Late-onset neonatal sepsis was significantly reduced with probiotics (RR 0.70, 95%CI 0.55-0.88), although moderate heterogeneity was observed due to differences in probiotic strains (Figure 4). Probiotics were also associated with shorter time to full enteral feeds (mean difference (MD) −2.5 days, 95%CI −4.0 to −1.0; I² ≈ 60%) (Figure 5) and reduced hospital length of stay (MD −3.0 days, 95%CI −5.0 to −1.0; I² ≈ 55%) (Figure 6).

**Figure 7:**
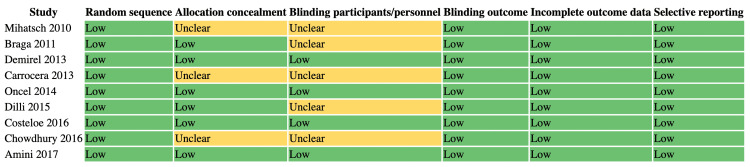
Traffic light–style risk of bias summary References: [16-24]

Risk of bias was visually summarized using a traffic light-style figure (Figure 7), with most studies showing low risk across domains, a few studies showing unclear allocation concealment or blinding, and one large multicenter trial having potential bias concerns. The summary of findings (Grading of Recommendations Assessment, Development and Evaluation (GRADE)) table (Table 5) indicates low certainty for mortality due to imprecision and inconsistency, and moderate certainty for sepsis, time to full feeds, and hospital stay, mainly due to heterogeneity across strains. Overall, these findings suggest that probiotics reduce sepsis and improve feeding and hospitalization outcomes, without a significant impact on mortality. 

**Table 5:** Summary of findings (GRADE) RR: risk ratio; GRADE: Grading of Recommendations Assessment, Development and Evaluation; MD: mean difference

Outcome	Assumed risk (control)	Effect size (95% CI)	Absolute effect	Certainty (GRADE)	Reasons for rating
Sepsis	20 per 100	RR 0.70 (0.55 to 0.88)	6 fewer per 100 (from 14 to 8)	Moderate	Inconsistency/indirectness
Mortality	10 per 100	RR 0.95 (0.80 to 1.12)	No important difference	Low	Imprecision/inconsistency
Time to full feeds	10 days	MD -2.5 days (-4.0 to -1.0)	2.5 days earlier	Moderate	Inconsistency (I2 ~60%)
Hospital stay	20 days	MD -3.0 days (-5.0 to -1.0)	3 days shorter	Moderate	Inconsistency (I2 ~55%)

Synthesis of findings and critical analysis of discrepancies

Probiotic Strain and Formulation

The conflicting outcomes, particularly regarding NEC, can be partly reconciled by considering the specific probiotic strains and formulations used. Studies that reported a significant reduction in NEC frequently utilized multi-strain formulations, such as the *B. breve* + *L. casei *combination in the trial by Braga et al. [17] and the multi-strain blends in the studies by Chowdhury et al. [23] and Amini et al. [24]. In contrast, several single-strain interventions, including *B. breve* in Costeloe et al. [22], *S. boulardii* in Demirel et al. [18], and *L. reuteri* in Oncel et al. [20], failed to demonstrate a significant effect on NEC. This dichotomy suggests that a synergistic or complementary effect between multiple strains may be necessary to achieve the desired clinical outcome of NEC prevention. The efficacy may not reside in a single "magic bullet" strain but rather in a balanced consortium that can more effectively modulate the gut environment and immune system.

Dosage and Intervention Duration

Another major factor contributing to the variability in results is the wide range of dosages and intervention durations. The studies reviewed here varied in dose from 6.6×105 CFU/g of *Streptococcus thermophilus* in Carrocera et al. (2013) [19] to 1.2×1010 CFU/kg/day of *B. lactis* in Mihatsch et al. [16]. Intervention durations also ranged dramatically, from a mere five days in Carrocera et al. [19] to eight weeks in Dilli et al. [21]. The fact that a very short five-day trial by Carrocera et al. [19] reported a positive outcome for NEC is a unique anomaly that underscores the complexity of this research. It is possible that there is a minimum effective dose or duration, but with such high variability, it is impossible to identify it from the current data. The lack of standardized protocols for both dosage and duration makes direct comparisons between trials extremely challenging and complicates the process of synthesizing the evidence. 

## Conclusions

Probiotics are a safe and effective intervention for preterm and VLBW infants. The study found that specific probiotic supplementation, particularly using multi-strain formulations, significantly reduces the incidence of NEC. It also improves feeding tolerance, helps infants reach full oral feeding sooner, and shortens their hospital stays. While probiotics demonstrated systemic anti-inflammatory benefits, they did not consistently affect mortality rates.

The findings underscore the clinical value of probiotics in this vulnerable population, emphasizing that the selection of specific strains is crucial for maximizing therapeutic outcomes. Therefore, future research must not only aim to identify the optimal, most efficacious strains and formulations but also strive to build a consensus on standardized approaches for clinical dosing, administration, and monitoring. Achieving this standardization is paramount for ensuring consistent, evidence-based care in the preterm setting.

## References

[1] (2025). World Health Organization: Preterm birth. https://www.who.int/news-room/fact-sheets/detail/preterm-birth.

[2] (2025). March of Dimes: A profile of prematurity in United States. https://www.marchofdimes.org/peristats/reports/united-states/prematurity-profile.

[3] Walani SR (2020). Global burden of preterm birth. Int J Gynaecol Obstet.

[4] Odira CC, Onyeje BT, Udeogalanya EA, Olabisi OI, Esan DT (2025). Predictors of mothers' home cord care, breastfeeding, and thermoregulation practices for newborns in a South-Eastern State, Nigeria. BMC Pregnancy Childbirth.

[5] Denning NL, Prince JM (2018). Neonatal intestinal dysbiosis in necrotizing enterocolitis. Mol Med.

[6] Van Belkum M, Mendoza Alvarez L, Neu J (2020). Preterm neonatal immunology at the intestinal interface. Cell Mol Life Sci.

[7] Sunil B, Bhavya S (2023). Efficacy of probiotics in preterm neonates in the prevention of necrotising enterocolitis: a randomised controlled trial. J Clin Diagn Res.

[8] Mercer EM, Arrieta MC (2023). Probiotics to improve the gut microbiome in premature infants: are we there yet?. Gut Microbes.

[9] Rogers AW, Tsolis RM, Bäumler AJ (2021). Salmonella versus the microbiome. Microbiol Mol Biol Rev.

[10] Singh DK, Miller CM, Orgel KA, Dave M, Mackay S, Good M (2022). Necrotizing enterocolitis: bench to bedside approaches and advancing our understanding of disease pathogenesis. Front Pediatr.

[11] Shen Y, Fan N, Ma SX, Cheng X, Yang X, Wang G (2025). Gut microbiota dysbiosis: pathogenesis, diseases, prevention, and therapy. MedComm (2020).

[12] Abdullahi AM, Zhao S, Xu Y (2025). Efficacy of probiotic supplementation in preventing necrotizing enterocolitis in preterm infants: a systematic review and meta-analysis. J Matern Fetal Neonatal Med.

[13] He P, Yu L, Tian F, Chen W, Zhang H, Zhai Q (2024). Effects of probiotics on preterm infant gut microbiota across populations: a systematic review and meta-analysis. Adv Nutr.

[14] Deshmukh M, Patole S (2021). Prophylactic probiotic supplementation for preterm neonates—a systematic review and meta-analysis of nonrandomized studies. Adv Nutr.

[15] Sharif S, Meader N, Oddie SJ, Rojas-Reyes MX, McGuire W (2023). Probiotics to prevent necrotising enterocolitis in very preterm or very low birth weight infants. Cochrane Database Syst Rev.

[16] Mihatsch WA, Vossbeck S, Eikmanns B, Hoegel J, Pohlandt F (2010). Effect of Bifidobacterium lactis on the incidence of nosocomial infections in very-low-birth-weight infants: a randomized controlled trial. Neonatology.

[17] Braga TD, da Silva GA, de Lira PI, de Carvalho Lima M (2011). Efficacy of Bifidobacterium breve and Lactobacillus casei oral supplementation on necrotizing enterocolitis in very-low-birth-weight preterm infants: a double-blind, randomized, controlled trial. Am J Clin Nutr.

[18] Demirel G, Erdeve O, Celik IH, Dilmen U (2013). Saccharomyces boulardii for prevention of necrotizing enterocolitis in preterm infants: a randomized, controlled study. Acta Paediatr.

[19] Fernández-Carrocera LA, Solis-Herrera A, Cabanillas-Ayón M, Gallardo-Sarmiento RB, García-Pérez CS, Montaño-Rodríguez R, Echániz-Aviles MO (2013). Double-blind, randomised clinical assay to evaluate the efficacy of probiotics in preterm newborns weighing less than 1500 g in the prevention of necrotising enterocolitis. Arch Dis Child Fetal Neonatal Ed.

[20] Oncel MY, Sari FN, Arayici S (2014). Lactobacillus reuteri for the prevention of necrotising enterocolitis in very low birthweight infants: a randomised controlled trial. Arch Dis Child Fetal Neonatal Ed.

[21] Dilli D, Aydin B, Fettah ND (2015). The ProPre-Save study: effects of probiotics and prebiotics alone or combined on necrotizing enterocolitis in very low birth weight infants. J Pediatr.

[22] Costeloe K, Hardy P, Juszczak E (2015). Bifidobacterium breve BBG-001 in very preterm infants: a randomised controlled phase 3 trial. Lancet.

[23] Chowdhury T, Ali MM, Hossain MM, Singh J, Yousuf AN, Yasmin F, Chowdhury FR (2016). Efficacy of probiotics versus placebo in the prevention of necrotizing enterocolitis in preterm very low birth weight infants: a double-blind randomized controlled trial. J Coll Physicians Surg Pak.

[24] Amini E, Dalili H, Niknafs N, Shariat M, Nakhostin M, Jedari-Attari S (2017). The effect of probiotics in prevention of necrotising enterocolitis in preterm neonates in comparison with control group. Innov J Ped.

